# Spatial Outcomes of Soft Tissue Sarcoma in Southern West Virginia

**DOI:** 10.7759/cureus.11454

**Published:** 2020-11-12

**Authors:** Frank H Annie, Christopher K Uejio, Sarah Embrey

**Affiliations:** 1 Cardiology, Charleston Area Medical Center, Charleston, USA; 2 Geography, Florida State University, Tallahassee, USA; 3 Pharmacy, University of Charleston School of Pharmacy, Charleston, USA

**Keywords:** dioxins, polychlorinated biphenyls (pcbs), k function

## Abstract

Introduction

Dioxins, polychlorinated biphenyls (PCBs), and 2,3,7,8-Tetrachlorodibenzodioxin (TCDDs) are persistent organic pollutants widely distributed in the food chain. For over 50 years, the Monsanto plant in Nitro, West Virginia, created dioxin waste while producing herbicides, Agent Orange (during the Vietnam War), and different forms of rubber. Recent and past literature has established a link between the Monsanto plant and increased cancer cases within the region. Soft tissue sarcoma is one of the few specific cancers that has been linked to dioxin exposure. This pilot study examined whether sarcoma cases were clustered or randomly distributed within Kanawha County, West Virginia over the years 2000 to 2013. We hypothesize that sarcoma cancer cases will be spatially clustered.

Methods

This study assessed the spatial distribution of cancer patients with addresses within the Nitro, West Virginia, and study area. The Charleston Area Medical Center shared soft tissue sarcoma (n = 97) cases from 2000 to 2013. An unweighted K function with confidence intervals (99 Monte Carlo permutations) and 10 distance at 2800 meters each band analyzed the distribution of cases.

Results

The results suggest that sarcoma cases are slightly clustered within the study area. The region also has a high concentration of chemical and industrial sites. The eighth distance band exhibited the greatest difference (11384), between the expected versus the observed K function.

Conclusion

The unweighted K function shows non-random clustering. Future studies could investigate possible associations to industrial, chemical, or other possible point source contamination within the study area.

## Introduction

Dioxins are a class of chemical by-products generated in various manufacturing processes, such as the production of rubbers and synthetic plastics. Dioxins are relatively stable chemicals that can degrade via sunlight and persist in the environment for several years [1]. Dioxin exposure research has been evolving since the first accident at the Monsanto plant in Nitro, West Virginia, in 1949. Subsequent accidents in West Germany in 1953 spurred more research into the long-term human health effects of dioxin exposure. From 1962 to 1970, the U.S. government commissioned companies such as Monsanto to produce Agent Orange, which created dioxin by-products; by the 1970s, the U.S. government had banned the use of the substance [1]. The primary avenue of human exposure is through contaminated foods [2]. Dioxins are divided into dioxin-like compounds and 2,3,7,8-Tetrachlorodibenzodioxin (TCDDs), which is one of the most toxic members of the dioxin family. Both dioxins and TCDDs are highly lipophilic and accumulate in human body fat [1].

Since 2003, the EPA and the National Academy of Sciences have attempted to improve the clarity of dose-response curves and the toxicity of these chemicals in the general population. Exposure models attempt to explain the range of dosages that cause negative effects on human health [3-5]. Animal testing and several human trials have provided a benchmark for exposure. These studies were conducted to evaluate the long-term reproductive and developmental conditions that could occur with exposure to TCDDs over an extended period. Dose-response curve studies revealed that greater exposure to TCDD and polychlorinated biphenyls (PCBs) increased the odds of adverse health outcomes [6], which range from cancers to forms of multigenerational epigenetic changes and heart and neurological disorders [7].

Various authors have concluded that the evidence regarding the chronic effects of 2,3,7,8-Tetrachlorodibenzo-p-dioxin (TCDD) exposures in humans is equivocal [8-10]. Cancer incidences are elevated in populations with relatively high exposures to TCDDs [11-12]. Studies on populations of concern have found increased rates of cancer among chemical workers and soldiers exposed to Agent Orange [3,8,11,13].

This study updates findings with several historical studies of accidental TCDD releases in 1949, 1962, and 1971 in Nitro, West Virginia [3,8,11,13].

Most cancer cluster studies do not find an association with a point source exposure (i.e., a contaminant or an industrial exposure site). However, it should be noted that only two studies confirm clustering of childhood cancer as a result of environmental contaminants; the two related sites are in Woburn, Massachusetts, and Dover Township, New Jersey [14]. The Woburn, Massachusetts, report examined six cases of childhood leukemia from 1969 to 1979. These cases were geographically clustered, and arsenic was theorized as the possible point source for leukemia cases. Geographic data analysis and spatial statistics investigated the relationship of cancer clusters in regions and possible sources.

Soft tissue sarcoma is an extremely rare cancer subtype that affects 6.26 men and 4.60 women per 100,000 per year. Over the 13-year study period, 60 cases were observed; this is above the national average of these cases in the same studied time frame [15]. This type of sarcoma is recognized as dioxin sensitive within the established literature [16-17].

## Materials and methods

This study assessed the spatial distribution of cancer patients with addresses in the Nitro, West Virginia, study area and all sarcoma patients recorded in the Charleston Area Medical Center and affiliated David Lee Cancer Center cancer registry from 2000 to 2013. The study excluded patients younger than 18 at diagnosis, which is a notable study limitation since some types of soft tissue sarcoma occur almost exclusively in pediatric populations [1]. The home billing address of each patient was geocoded using ArcGIS 10.6 to latitude and longitude. The ESRI Street Map geocoder reported an accuracy of 100%.

This study examines whether soft tissue sarcoma cases are clustered compared to complete spatial randomness. An unweighted Ripley’s K function with a maximum distance band of 26 miles quantified the degree of spatial clustering. In the equation, K (h) is the expected number of events inside the radius (h). The area is described as A, and the number of observed events is described as N. The distance function is illustrated as dij, which is the distance between the events of i and j. The K function did not artificially adjust for edge corrections which set wi to 1. Monte Carlo simulations created a 99% null confidence envelope, using 99 permutations as confidence bands.

## Results

**Figure 1:**
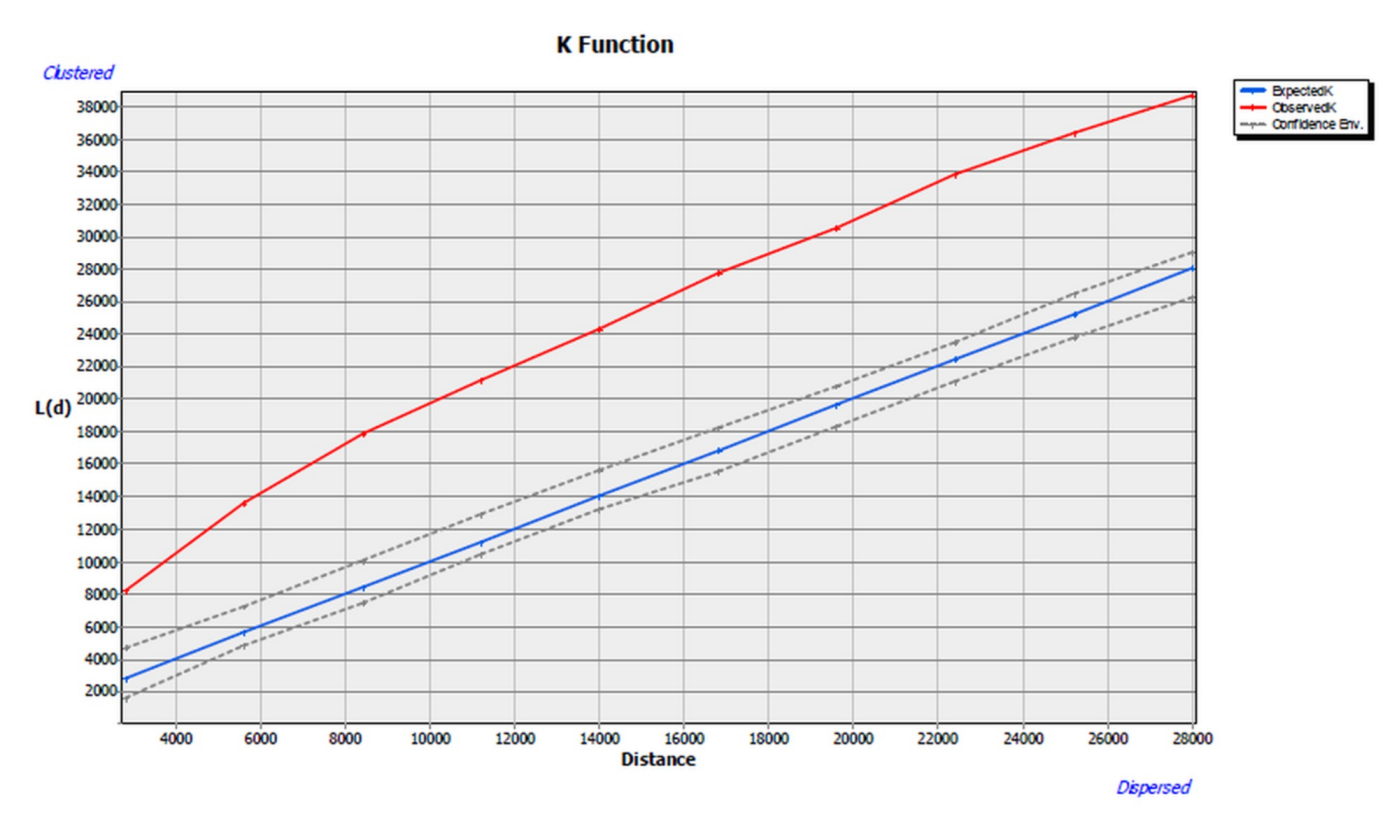
K-Function Graphic

Table 1 and Figure 1 present the K function relationship based on the 10 distance bands. The observed K was significantly above the expected K across all 10 distance bands. The eighth distance band exhibited the largest difference (11384), between the expected versus the observed K function. Although evidence seems to support the occurrence of clustering within the Nitro and Saint Albans areas, further epidemiological and spatial studies are required. As this is designed as a pilot study, the supposed distribution of soft tissue sarcoma may be influenced by other factors that were not directly studied in the Charleston Area Medical Center service area.

**Table 1:** Output of Ripley’s K-Function

OBJECTID *	ExpectedK	ObservedK	DiffK	LwConfEnv	HiConfEnv
1	2800.77355	8143.252776	5342.479226	1578.908991	4630.270075
2	5601.5471	13545.530075	7943.982975	4789.068085	7200.932918
3	8402.320651	17835.382795	9433.062145	7405.739613	10035.70254
4	11203.094201	21124.362583	9921.268382	10401.643441	12904.623223
5	14003.867751	24255.66016	10251.792409	13172.303163	15582.479928
6	16804.641301	27709.740328	10905.099026	15502.281352	18222.5525
7	19605.414852	30485.62124	10880.206388	18277.19296	20791.299968
8	22406.188402	33790.127984	11383.939582	21065.273341	23450.918198
9	25206.961952	36342.355698	11135.393746	23746.707235	26476.754947
10	28007.735502	38668.767368	10661.031865	26192.76295	28993.515727

## Discussion

Our study suggests that there might be a suggestive relationship of clustering within the service area of the Charleston Area Medical Center. Studies such as this are necessary to understanding whether there is any association between soft tissue sarcoma and point source contamination. Soft tissue sarcoma represents less than 1% of total cancers diagnosed per year [15, 18]. Previous studies have supported a connection between soft tissue cancers and dioxin-based exposure [16-17]. Other studies in the area have established relationships between dioxin and low birth weight as an effect of production of dioxin-based exposure from 1955-1969 [19]. Exposure and production occurred decades prior to this study that could establish a possible continued environmental contaminant. Further research in the region has established a possible spatial relationship to congenital heart defects as a result of chemical and coal extraction sites [20]. Point source contamination as described in this study describes a statistical significant relationship to potential points of concern in the region in relation to congenital heart defects. Other disease states have also been explored as well such as Müllerian anomalies within the obstetric population in the same region [21]. The obstetric population showed a similar statistical relationship between Müllerian anomalies and EPA regulated facilities. Over all literature in the region shows a suggestive relationship with differing point source contamination and possible exposure which requires further research.

## Conclusions

Based on the findings of this study, more research is necessary. Moreover, many confounders may not be represented in the data. The data’s accuracy was also limited to the documentation in the electronic health records. As the half-life of dioxin is over 11 years, the most impactful future direction for this project would be to analyze blood samples from the 97 individual cases to quantitatively determine their exposure to dioxins. This testing could more definitely link dioxin exposure to the occurrence of these cancers. Once this information is gathered, a more thorough spatial analysis can be conducted to evaluate and explain any potential variables and establish controls within the population, which could assist in verifying environmental causality.
